# The role of suPAR and related proteins in kidney, heart diseases, and diabetes

**DOI:** 10.1172/JCI197141

**Published:** 2026-01-02

**Authors:** Jochen Reiser, Salim S. Hayek, Sanja Sever

**Affiliations:** 1 Department of Internal Medicine and; 2 Sealy Institute for Drug Discovery, University of Texas Medical Branch, Galveston, Texas, USA.

**Keywords:** Cardiology, Immunology, Nephrology, Chronic kidney disease, Innate immunity

## Abstract

The urokinase plasminogen activator receptor (uPAR) is a membrane-bound protein found on the surface of immune cells. Through the action of proteases, uPAR is cleaved to produce several circulating proteins in the bloodstream, including the soluble form suPAR and the fragments D1 and D2D3. Initially studied in the context of infectious diseases and cancer, recent research has revealed roles for suPAR and its related proteins as mediators linking innate immunity to the pathogenesis of kidney and cardiovascular diseases, as well as insulin-dependent diabetes. While these proteins have long been recognized as prognostic biomarkers, growing clinical, experimental, and genetic evidence highlights their active involvement in the onset and progression of these diverse conditions. This Review examines suPAR’s evolution from its discovery as a modulator of innate immunity to its current status as a key driver in chronic kidney and cardiovascular diseases. Furthermore, we explore the molecular mechanisms through which suPAR and D2D3 contribute to multiorgan damage, emphasizing emerging opportunities for therapeutic interventions across interconnected organ systems.

## Abstract 2

The uPAR system, particularly its soluble forms such as suPAR and D2D3, has emerged as a key mediator linking inflammation, immunity, and coagulation.

## Innate immunity, uPAR, and its associated circulating proteins

The innate immune system is the first line of defense against pathogens, responding rapidly through mechanisms that do not require prior exposure. Innate immunity involves the production of proinflammatory cytokines, chemokines, and antimicrobial peptides, as well as the recruitment and activation of immune cells to the site of infection or injury (1). Overactive or dysregulated innate immune responses resulting in chronic inflammation are central features of numerous disorders such as autoimmune diseases (2), cancer (3), diabetes mellitus (4, 5), kidney disease (6), and cardiovascular (CV) disease (7).

Central contributors to chronic inflammation include TLRs, various interleukins (IL-6, IL-1, IL-2), chemokines, TNF-α, IFN-γ, NLRP3 inflammasomes, and dysregulated complement, which have all been explored as potential therapeutic targets with varying degrees of success. Alongside these classic biomarkers of chronic inflammation, the urokinase plasminogen activator receptor (uPAR) and its soluble form (suPAR) have also attracted interest due to their link to innate immunity.

**Figure 1:**
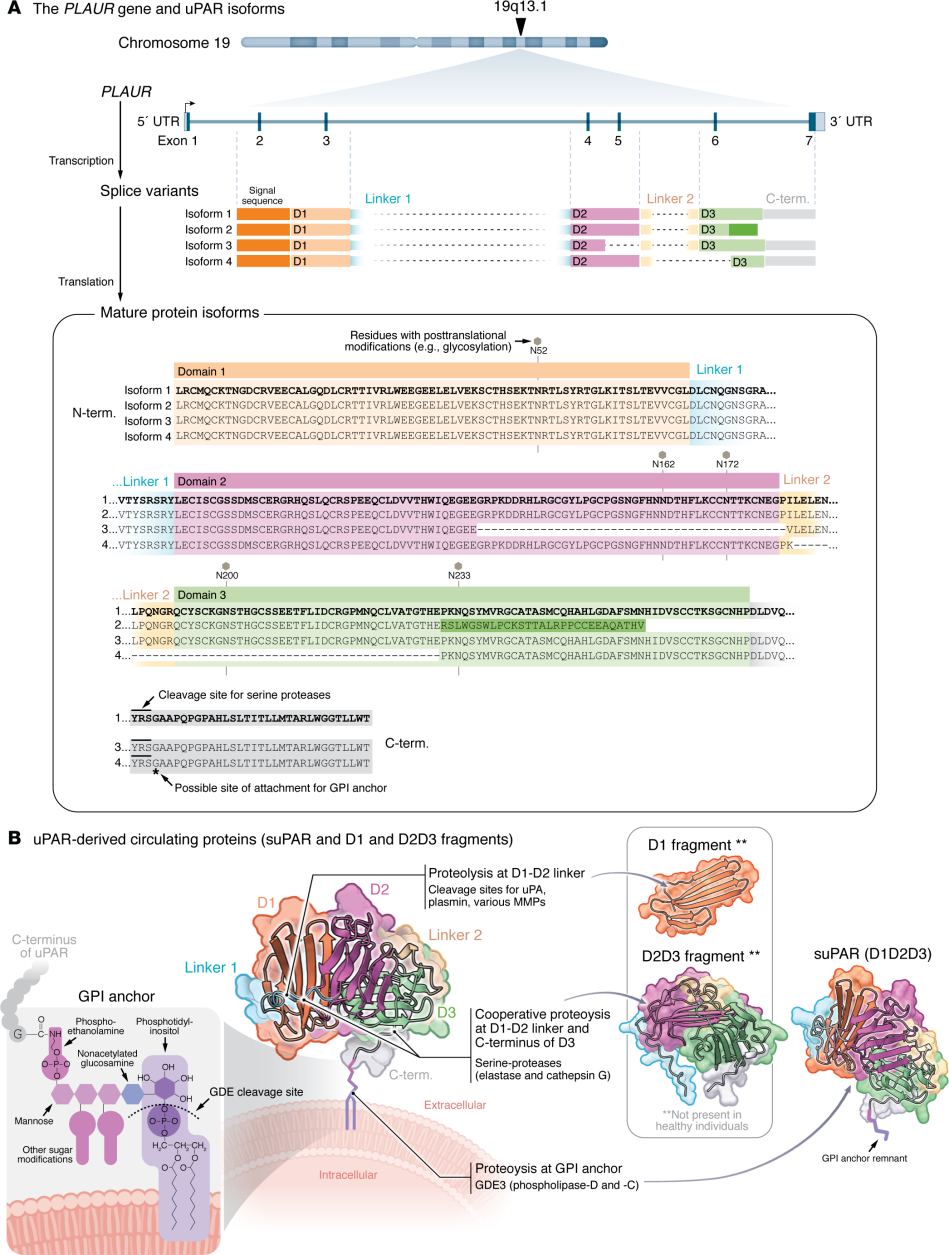
Posttranscriptional processing and proteolysis work together to produce multiple uPAR protein variants. (**A**) *PLAUR*, located on chromosome 19, produces multiple isoforms of uPAR through posttranscriptional processing. Isoform 1 is commonly known as uPAR. The nascent uPAR protein contains a short peptide at the N-terminus, which is crucial for targeting the protein to the secretory pathway. This sequence directs the ribosome-protein complex to the endoplasmic reticulum (ER), ensuring proper folding and posttranslational modifications, such as glycosylation and the addition of a glycosylphosphatidylinositol (GPI) anchor at the C-terminus. Once inside the ER, this signal sequence is cleaved by signal peptidase, leaving the mature protein to undergo further processing and localization at the plasma membrane. The glycosylation sites on asparagine (N) are indicated accordingly in the figure. Within the ER, the GPI transamidase recognizes a specific sequence at the protein’s C-terminus, cleaves the peptide bond, and transfers the GPI anchor to the glycine residue. Isoforms 1, 3, and 4 are GPI-anchored proteins, whereas isoform 2 lacks the GPI anchor and is directly secreted into circulation. (**B**) uPAR isoform 1 comprises three homologous domains: domain 1 (D1), D2, and D3. uPAR can be released from the plasma membrane into circulation through the action of multiple proteases. Proteolysis at the GPI anchor by phospholipase C (PLC), phospholipase D (PLD), or glycophosphodiesterase 3 (GDE3) generates circulating soluble uPAR (suPAR), consisting of D1, D2, and D3. Urokinase plasminogen activator (uPA), plasmin, and several matrix metalloproteinases (MMPs) predominantly cleave within the linker region between D1 and D2, releasing D1. These proteolytic events can indirectly facilitate the release of D2D3 or suPAR by exposing the GPI anchor to additional enzymes. Cooperative proteolysis by elastase and cathepsin G has been implicated in the formation of the D2D3 fragment. All structural models of UPAR_Human (UniProt accession number Q03405) were generated using AlphaFold version 2 (111).

The *PLAUR* gene encodes a pre-uPAR protein with an N-terminal signal peptide consisting of the first 22 amino acids, essential for directing the nascent protein into the secretory pathway for processing and cell surface transport (Figure 1A). There are 16 predicted splice variants of *PLAUR*. Isoform 1, the most prevalent, is the primary focus in human studies and is referred to as uPAR in this text (Figure 1A).

uPAR is a multiligand receptor that lacks transmembrane and intracellular domains and is instead loosely attached to the cell membrane via a C-terminal glycosylphosphatidylinositol (GPI) anchor. Along with GPI anchoring, uPAR undergoes posttranslational glycosylation, which increases its molecular weight from 35 kDa to an apparent 55 kDa when analyzed by SDS-PAGE. Structurally, uPAR consists of three extracellular domains (D1, D2, and D3), each adopting a three-finger protein fold composed of three β-stranded loops stabilized by disulfide bonds, thus resembling a hand with three fingers (Figure 1B). This three-finger fold, characteristic of the Ly-6/uPAR/α-neurotoxin protein family, enables high-affinity interactions with diverse molecular targets. Notably, some members of this family are neurotoxins found in snake venoms (8–10). Common ligands for uPAR include urokinase-type plasminogen activator (uPA), vitronectin, integrins, and the formyl (fMLF) family peptides. Through these interactions, uPAR regulates cell adhesion, migration, and proteolysis, influencing processes such as wound healing, immune response, and cancer metastasis (11).

Multiple proteases can convert uPAR into distinct circulating proteins (12, 13) (Figure 1B). As uPAR is tethered to the membrane by a GPI anchor, a majority of suPAR is generated by GPI-anchor cleavage catalyzed by members of the glycerophosphodiesterase 3 (GDE3) family, such as secreted phospholipase D or membrane-associated phospholipase C (14, 15).

Moreover, the D1-D2 linker region of uPAR is particularly vulnerable to proteolytic cleavage by several enzymes, including uPA (16), plasmin, and a range of matrix metalloproteinases (MMPs) (17). In addition, serine proteases such as neutrophil elastase and cathepsin G can act synergistically to release the D2D3 fragment from monocytic cells (18). Although both enzymes target the D1-D2 linker, cathepsin G also exhibits a strong ability to cleave the C-terminal end of the D3 domain (Figure 1B). As neutrophils release both proteases, this process is especially relevant in inflammatory conditions where neutrophil elastase activity is high. Indeed, unlike suPAR, D1 and D2D3 proteins are not found in healthy individuals but have been detected in the serum of patients with cancer and kidney disease (19, 20). Although they are structurally distinct (Figure 1B), all uPAR-related circulating proteins (suPAR, D1, and D2D3) have been implicated in immune signaling pathways (21).

## uPAR and associated proteins as modulators of physiology

uPAR is expressed across various cell types, with its expression levels often linked to specific physiological conditions. For instance, in immune cells, uPAR is involved in immune signaling, cell migration, and chemotaxis; in endothelial cells, it responds to injury or inflammation; in fibroblasts, it aids in tissue repair and extracellular matrix (ECM) remodeling; in epithelial cells, it supports tissue remodeling and repair; and in adipocytes, it contributes to metabolic dysfunction and ECM remodeling, affecting fat tissue plasticity and structure (11). The primary sources of uPAR/suPAR include the bone marrow (22) and immune cells such as monocytes-macrophages (23), neutrophils (24), and dendritic cells (25).

Membrane-bound uPAR is best known for its key role in regulating the plasminogen activation system by binding uPA, a serine protease. uPA binding to uPAR catalyzes the conversion of inactive plasminogen into active plasmin, leading to the degradation of ECM proteins like fibrin (26). As this pathway is central in mediating proteolysis during cancer invasion and metastasis, therapeutic strategies that target uPA and its receptor in cancer have been explored (27–29).

**Figure 2:**
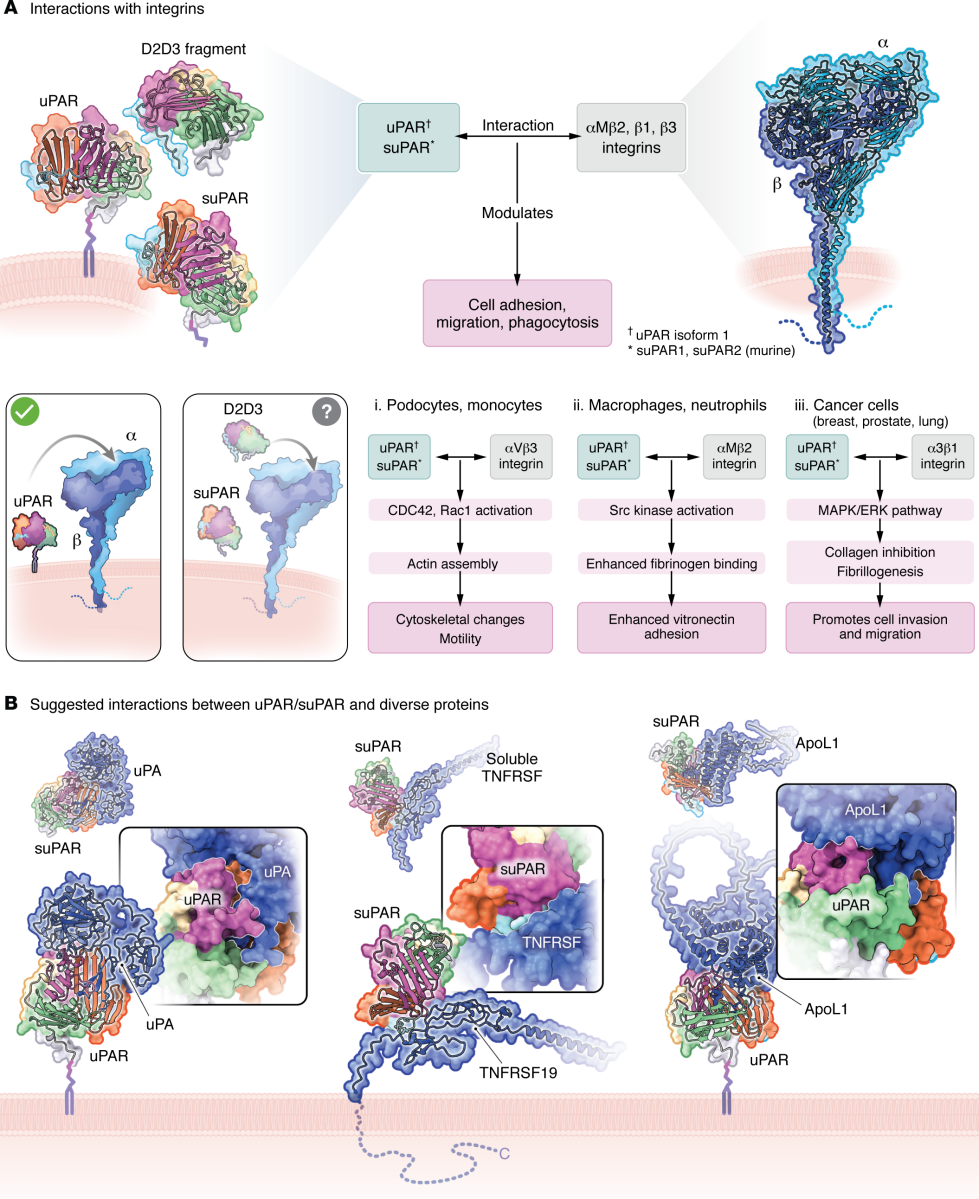
uPAR and its associated proteins engage in a variety of protein-protein interactions. (**A**) Interactions with integrin: Downstream pathways activated by interactions between integrins and uPAR or its related proteins, suPAR and D2D3, across different cell types. All structural models were generated using AlphaFold version 2 (111). UniProt numbers: UPAR_HUMAN (Q03405), ITAV_HUMAN (P06756), and ITB3_HUMAN (P05106). suPAR and αvβ3 integrin on podocytes: The activation of αvβ3 integrin on podocytes by uPAR and suPAR has been proposed as a mechanism contributing to podocyte injury. suPAR and αMβ2 on macrophages and neutrophils: uPAR binding to αMβ2 integrin facilitates the adhesion of macrophages and neutrophils to ECM components and endothelial cells. uPAR/suPAR and αMβ2 integrin interactions influence cytoskeletal rearrangements and reactive oxygen species (ROS) production in those cells. suPAR and α3β1 integrin on cancer cells (breast, prostate, and lung): While direct interactions between uPAR and the β1 integrin subunit have been observed in various cell types, such as fibroblasts, macrophages, and monocytes, this interaction is particularly well studied in cancer cells. In this context, direct uPAR–α3β1 integrin interactions play a critical role in promoting tumor cell migration, invasion, and metastasis. (**B**) Suggested interactions between uPAR/suPAR and diverse proteins: Computer models were generated using AlphaFold version 2 (111) and protein information from UniProt (https://www.uniprot.org/). uPAR and uPA UniProt numbers: UPAR_HUMAN (Q03405) and UROK_HUMAN (P00749). When uPA binds to membrane-associated uPAR, it forms a complex that facilitates the conversion of plasminogen to plasmin. Moreover, uPA can cleave uPAR itself, resulting in the release of suPAR from the cell surface. suPAR and TNFRSF19 UniProt numbers: UPAR_HUMAN (Q03405) and TNR19_HUMAN (Q9NS68). TNFRSF19 is a TNF receptor superfamily member, a transmembrane protein implicated in inhibiting the TGF-β signaling pathway. suPAR and ApoL1 UniProt numbers: UPAR_HUMAN (Q03405) and APOL1_HUMAN (O14791). This interaction has been implicated in inducing chronic kidney diseases, particularly in individuals of recent African ancestry.

In addition, uPAR binds vitronectin, a major ECM component, promoting the initial attachment of cells to the matrix (30), as well as to various integrins, transmembrane proteins that play crucial roles in cell adhesion, signaling, and communication with the ECM (Figure 2A). SuPAR and D2D3 share some of these interactions, as integrin binding sites have been identified within the D2 and D3 domains (31). While integrins are expressed ubiquitously, integrin subtypes and activation thresholds are regulated in a cell-type-specific manner (32). Integrins that are preferentially recognized by uPAR and its related circulating proteins include αvβ3, αMβ2, and α3β1 (9, 33).

Activation of αvβ3 integrin on podocytes and monocytes by uPAR or suPAR has been linked to disruptions in actin cytoskeleton dynamics and motility through Cdc42/Rac1-mediated actin polymerization, which can lead to podocyte injury (34, 35) (Figure 2A, schematic). Podocyte injury ultimately results in chronic kidney disease (CKD) across various patient populations (33–36), while suPAR-activated monocytes have been implicated in atherosclerosis (37). In macrophages and neutrophils (Figure 2A, schematic ii), the uPAR-αMβ2 complex activates Src kinases, promoting fibrinogen binding and vitronectin adhesion, thereby influencing cellular adhesion, migration, and immune responses (38). These mechanisms enhance the ability of cells to migrate toward sites of inflammation or injury.

Direct interactions between uPAR and the β1 integrin subunit have been observed in cell types such as fibroblasts, macrophages, and monocytes, with a particularly well-characterized role in cancer cells (Figure 2A, schematic iii). Indeed, uPAR-α3β1 integrin interactions are crucial for tumor cell migration, invasion, and metastasis via activation of the MAPK/ERK pathway (9, 39, 40). Although uPAR/suPAR primarily function as monomers, dimerization has been observed on the protein level and the cell surface, with a crystal structure identifying a major conformational change within the dimer compared with the monomer (41), further expanding mechanisms by which these proteins influence cell physiology.

While uPAR and its associated proteins do not directly bind TLRs, they can influence TLR signaling by interacting with integrins. These indirect interactions help amplify immune responses by modulating the production of inflammatory cytokines like TNF-α, IL-6, and IL-1β. Although early studies focused on uPAR-integrin interactions, it is now evident that suPAR and D2D3 interactions with integrins mediate similar cellular processes via distinct mechanisms. Elucidating these mechanisms has been essential for understanding tumor biology (19, 21, 42) and, more recently, kidney diseases (33–36).

## Significance of suPAR concentrations in kidney diseases

Although suPAR is present in the blood of healthy individuals, bone marrow–derived immature myeloid cells (22, 43, 44) and neutrophils appear to be the main source of suPAR during conditions of acute immune activation (22, 43, 44). Therefore, circulating suPAR concentrations are thought to reflect the status of innate immunity (45).

The link between suPAR concentrations and the status of the innate immunity is not simple. For example, even though tar and nicotine are known to suppress innate immune defenses, cigarette smoking itself is a potent stimulator of circulating suPAR levels. On average, smokers exhibit suPAR concentrations approximately 30% higher than non-smokers (3.3 ng/mL versus 2.1 ng/mL). Encouragingly, these elevated levels begin to decline within four weeks following smoking cessation (46). Beyond smoking, some acute infections are known to increase suPAR levels in the bloodstream. This is particularly evident in RNA viral infections, including HIV and SARS-CoV-2, as well as some bacterial infections (47–49). In contrast, under stable, non-acute conditions, an individual’s suPAR levels remain remarkably consistent, typically fluctuating by less than 10% over five years (50). Additionally, suPAR levels are not subject to daily circadian variations, nor are they significantly altered by certain inflammatory events like acute myocardial infarction or coronary artery bypass grafting (51, 52).

Although suPAR levels are consistent in healthy populations, a major challenge in establishing suPAR as a meaningful biomarker for CKD patients is that reduced kidney function often increases levels of proteins in circulation. For instance, β-2 microglobulin (β2M), cystatin C, and uremic toxins accumulate in the blood as kidney function declines (53–55). Similarly, inflammatory cytokines like IL-6 and C-reactive protein (CRP) are elevated in CKD due to reduced renal clearance and heightened inflammatory signaling (56, 57). Even though suPAR levels might rise with worsening kidney function, high suPAR levels have been shown to be a valuable biomarker for identifying individuals at risk of developing kidney disease and for predicting how quickly their condition may deteriorate (33, 58–62), including children ages 1–16 years (63).

To understand how elevated suPAR concentrations contribute to CKD, it was essential to begin by measuring their levels in people without kidney diseases. In a study of 3,683 patients undergoing coronary angiography, those in the highest suPAR quartile experienced an annual estimated glomerular filtration rate (eGFR) decline of –4.2 mL/min/1.73 m², compared with –0.9 mL/min/1.73 m² in patients in the lowest quartile. In addition, patients with an initial eGFR above 60 mL/min/1.73 m² faced a 3.13-fold increased risk of progressing to CKD stage 3, and adding suPAR concentrations to a multivariable model improved risk discrimination (ΔC-statistic of 0.08) (58). Furthermore, suPAR was the only biomarker, among others such as high-sensitivity CRP (hsCRP), that was associated with the decline in kidney function (59). These original findings were replicated in other non-CKD cohorts, including the Swedish Malmö Diet and Cancer Study (5,381 individuals) (62). Similar trends were also observed in the CKD-specific cohorts, including the African American Study of Kidney Disease and Hypertension (AASK) cohort (607 individuals) (33, 60). A study of patients with sickle cell disease (SCD) also showed that plasma suPAR concentrations positively correlated with stages of CKD (64). Elevated suPAR concentrations in the SCD population were attributed to the combination of peripheral blood mononuclear cells (PBMCs), activated macrophages, and increased levels of secreted phospholipase D and uPA. In all those studies, the association between elevated suPAR concentrations and declining kidney function persisted independently of traditional risk factors, such as age, race, baseline eGFR, diabetes, hypertension, or proteinuria.

Further evidence for suPAR’s role in kidney health emerged from a genome-wide association meta-analysis of over 24,000 individuals, which identified a missense variant in the *PLAUR* gene (rs4760) associated with higher suPAR concentrations (37). Subsequent Mendelian randomization analyses of the UK Biobank dataset, using the genetic variant rs4760, indicated a causal association between genetically predicted suPAR concentrations, atherosclerotic phenotypes, and CKD, further implicating suPAR in both conditions (37).

While these studies collectively provide compelling evidence for the role of suPAR in kidney health, the nephrology community remains divided on the value of measuring suPAR levels in the CKD population. Some of the skepticism stems from the fact that loss of kidney function contributes to increased suPAR levels in patients with CKD (65, 66). That said, as suPAR is not primarily cleared by the kidneys like creatinine or cystatin C, the relationship between suPAR levels and kidney function is complex and not purely due to reduced filtration. Current data suggest that suPAR levels are more influenced by increased production (from immune and endothelial cells) than by impaired renal function (21, 67). However, in advanced CKD, as kidney function declines severely (e.g., eGFR < 30 mL/min/1.73 m²), suPAR levels can rise further, likely due to a combination of increased systemic inflammation, impaired clearance, and tissue injury (68).

Furthermore, some of the skepticism stems from the methods used to measure suPAR concentrations (69). Currently, no FDA-approved assay exists for measuring suPAR concentrations. In research laboratories, suPAR is typically measured using ELISA kits, such as the human uPAR Quantikine ELISA kit from R&D Systems, and the suPARnostic ELISA or Quick Triage from Virogates, whose values we use in this Review. Commercially available ELISAs can detect both suPAR and D2D3 with similar sensitivity (20), complicating the clinical diagnostic value of suPAR concentrations when D2D3 is also present. Notably, D2D3 has thus far been detected only in patients with cancer (70–72), and kidney diseases (20). Since ELISAs cannot distinguish between suPAR and D2D3, detecting D2D3 relies on immunoprecipitation followed by Western blot analysis (20), a technique limited to specialized research laboratories. suPAR concentrations can also be determined using the aptamer-based SomaLogic SOMAscan (73) and the Olink Explore proximity extension assay (74). However, these assays show a weak correlation with ELISA-derived concentrations, likely because they detect protein fragments that include suPAR as well as D1 and D2D3 (75).

The variability in assay methodologies, along with suPAR’s association with conditions such as infections and declining kidney function, limits the specificity and sensitivity of suPAR levels as a standalone marker of kidney injury. Thus, beyond defining indication-specific cutoffs using standardized assays, it may be advantageous to integrate suPAR measurements with additional biomarkers or functional bioassays. Recent insights suggest that combining suPAR levels with the detection of nephrin (76) or CD40 autoantibodies (77) in patients with focal segmental glomerulosclerosis (FSGS) could be particularly valuable, aiming to develop a kidney disease–specific composite scoring system. Establishing standardized, FDA-approved assays for accurately quantifying suPAR and D2D3, alongside such composite scoring systems incorporating disease-specific biomarkers or bioassays, is expected to significantly enhance clinical utility. This approach would address the heterogeneity within the CKD population, leveraging the robust association of suPAR levels with key clinical outcomes, including kidney disease onset and progression, cardiovascular events, and mortality.

## Mechanisms of kidney injury

In 2008, it was proposed that uPAR activated αvβ3 integrin on the surface of podocytes, triggering a signaling cascade that activated canonical small GTPases Cdc42 and Rac1, which in turn led to dysregulation of the actin cytoskeleton, foot process effacement, and ultimately proteinuria (34) (Figure 2A). Mice lacking the *Plaur* gene (*Plaur^–/–^* mice) were protected from LPS-induced proteinuria but developed disease when expressing a constitutively active β3 integrin. This mechanism for podocyte injury was later extended to include suPAR (35) and D2D3 (20) (70). However, the role of suPAR as a nephrotoxic protein that directly harms podocytes by activating αvβ3 integrin has been challenged by murine models that have not exhibited any measurable phenotypes, including kidney-related phenotypes, despite high concentrations of suPAR in circulation (78, 79). Indeed, the onset of proteinuria and deterioration of kidney function in all currently available murine models requires the introduction of a second nephrotoxic insult, such as a high-fat diet (20) or an anti-CD40 autoantibody (80).

Similarly, studies have demonstrated that suPAR binds to apolipoprotein L1 (ApoL1), a circulating protein whose genetic variants, G1 and G2, are associated with an increased risk of kidney disease in individuals of recent African descent (33, 81–87). Evidence suggests that suPAR can form a tripartite complex with ApoL1 risk variants and αvβ3 integrin on podocytes, thus enhancing podocyte injury (33). The observed interactions between suPAR and ApoL1 variants provide a molecular framework for understanding how genetic risk factors for kidney disease interact with innate immune activation to accelerate nephropathy (33). Together, these murine models suggest that while a high concentration of circulating suPAR is insufficient to cause kidney damage by itself, it becomes a significant nephrotoxic factor in the presence of additional risk factors (ApoL1, anti-CD40 antibodies, high-fat diet), highlighting the multifactorial nature of CKDs. An exception to this rule is murine suPAR isoform 2 (Figure 1A), a protein variant directly secreted from cells that forms dimers in vitro (36). Transgenic expression of mouse isoform 2 independently induced severe kidney disease resembling FSGS, characterized by pronounced proteinuria and foot process effacement (36). Notably, the absence of β3 integrin in this transgenic model mitigated the kidney phenotype, emphasizing the pivotal role of integrin signaling in suPAR-mediated kidney injury. However, the relevance of this murine isoform 2, or any other *PLAUR*-encoded isoforms, to human kidney diseases remains to be determined.

A major challenge in uncovering the molecular mechanisms through which uPAR and its related proteins contribute to organ damage lies in their dual role as both receptors and ligands for a wide range of other proteins. Fortunately, recent advances in artificial intelligence tools, such as AlphaFold, have made it possible to predict specific protein-protein interactions with remarkable accuracy. For example, AlphaFold successfully identified extensive interactions between uPA and its receptor uPAR (Figure 2B), underscoring the robustness of modern computational methods. Beyond validating known interactions, AlphaFold also predicted novel interactions, such as those between suPAR and the cytoplasmic domain of TNFRSF19, a member of the TNF receptor superfamily (Figure 2B). As TNFRSF19, a transmembrane protein involved in inhibiting TGF-β signaling, has a circulating form linked to end-stage renal disease in patients with type 1 diabetes (88), these newly identified interactions seem worth pursuing. Furthermore, and in line with experimentally determined high-affinity binding (*K*_d_ ≈ 10 nM) using recombinant proteins (33), AlphaFold also predicted an extensive interface between suPAR and ApoL1 (Figure 2B). This limited analysis suggests that the advancements in computational tools may drive significant progress by generating innovative and testable hypotheses.

## Beyond CKD: AKI and insulin-dependent diabetes

**Figure 3:**
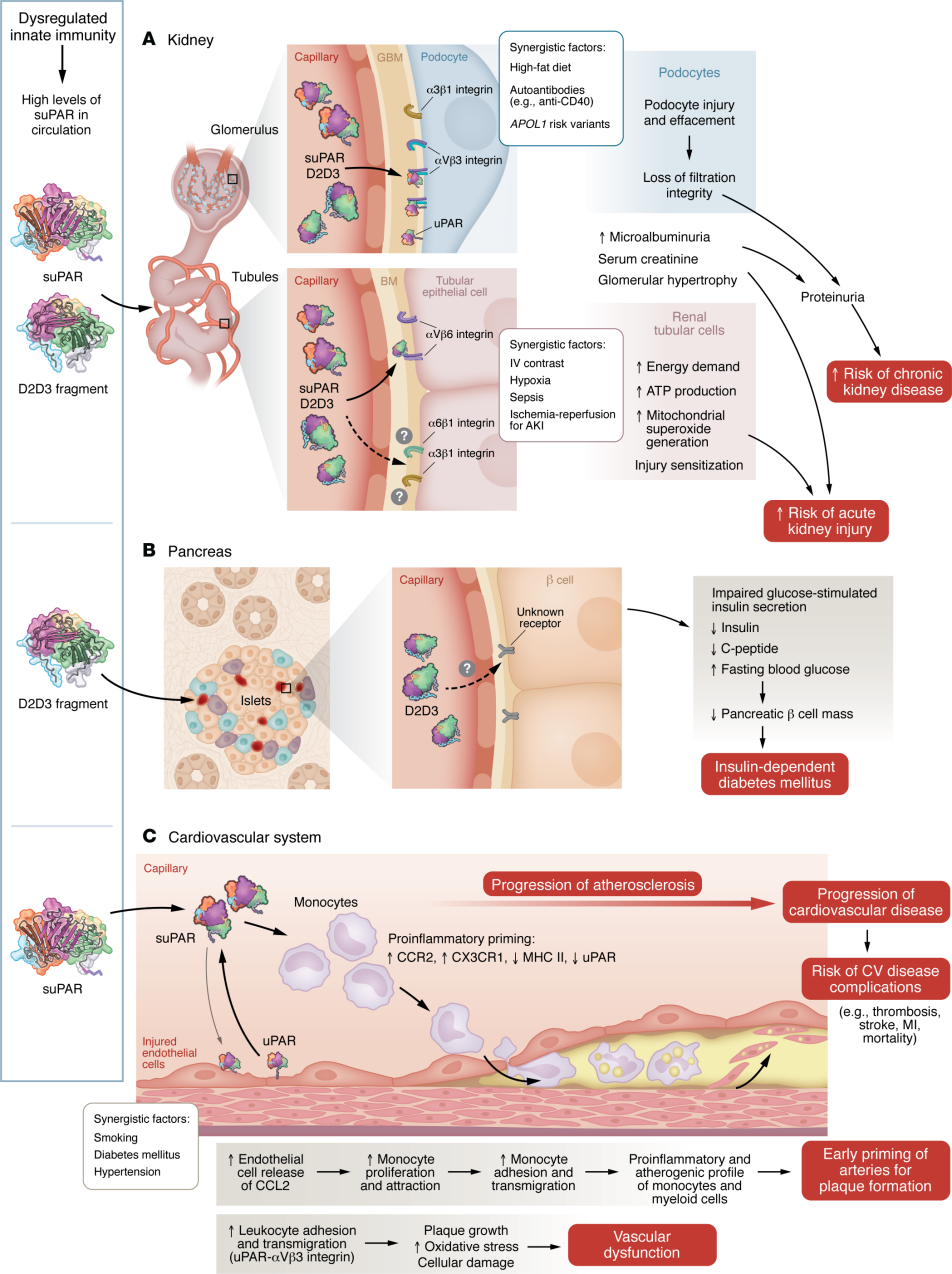
uPAR and its associated proteins induce multiorgan injury. Dysregulation of innate immunity caused by various physiological challenges, such as diabetes, hypertension, viral and bacterial infections, or smoking, leads to elevated suPAR levels and/or production of the D2D3 protein. Models illustrate the mechanisms through which these proteins cause injury to the kidney (**A**), pancreas (**B**), and cardiovascular system (**C**).

While the suPAR system has predominantly been investigated in the context of suPAR-αvβ3 integrin interactions on glomerular podocytes, several studies suggested that suPAR might also affect the metabolism of renal tubular epithelial cells (Figure 3A). In immortalized human proximal tubular cells (HK-2) in culture, suPAR increased ATP production, heightened energetic demand, and increased mitochondrial superoxide generation (89). While this metabolic shift may initially seem adaptive, the increased workload appears to sensitize proximal tubular cells to injury, potentially explaining the heightened risk of acute kidney injury (AKI) observed in clinical settings, including contrast-induced nephropathy, cardiac surgery, and sepsis. In a study of 3,827 patients undergoing coronary angiography (that were originally used to examine the link between suPAR and CKD), 250 patients undergoing cardiac surgery, and 692 critically ill patients, suPAR concentrations were measured to determine the relationship to AKI (77). AKI developed in 8% of those who had coronary angiography. Patients in the highest suPAR quartile had 2.66-fold higher adjusted odds of AKI and 2.29-fold higher odds of AKI or death at 90 days compared with those in the lowest quartile. Similar associations were also observed in patients undergoing surgery and in those who were critically ill. Furthermore, suPAR-overexpressing mice exposed to contrast material exhibited more severe functional impairment and histologic signs of AKI compared with wild-type mice (89).

A similar analysis of longitudinal serum suPAR concentrations identified disease severity and progression of sepsis-induced AKI in 200 critically ill patients meeting Sepsis-3 criteria (90). Patients with suPAR levels above 12.7 ng/mL faced the highest risk for renal replacement therapy (RRT) and death, with an adjusted odds ratio of 7.48 (95% CI 3.00–18.63). suPAR was suggested to induce kidney damage by promoting T cell infiltration; as in mice, suPAR deficiency protected against sepsis-induced AKI (90). Taken together, these studies establish the role of uPAR and related proteins in diverse types of CKD, all linked by the common theme of chronically activated innate immunity.

Adding further complexity to the molecular mechanisms by which suPAR contributes to kidney disease, D2D3 has been implicated in both pancreatic β cell dysfunction and kidney injury (20).

Although D2D3 alone did not directly induce kidney injury, mice overexpressing D2D3 and fed a high-fat diet developed progressive kidney disease characterized by microalbuminuria, elevated serum creatinine, and glomerular hypertrophy. Unexpectedly, these mice also developed insulin-dependent diabetes mellitus, as indicated by reduced insulin and C-peptide levels, decreased pancreatic β cell mass, impaired glucose-stimulated insulin secretion, and elevated fasting blood glucose, even in the absence of a high-fat diet. D2D3 by itself inhibited glucose-stimulated insulin release by pancreatic β cells in culture or from human islets (Figure 3B). Cell culture studies suggested that the binding of D2D3 to as yet unidentified receptors inhibited multiple cellular processes induced by high glucose levels in pancreatic β cells, including the maturation and trafficking of secretory vesicles and dysregulation of the cytoskeleton. As D2D3 was found predominantly in patients with diabetic nephropathy linked to insulin-dependent diabetes, this study suggested a molecular mechanism for the frequent cooccurrence of diabetes mellitus and kidney disease (42).

Notably, D2D3 was first detected in plasma from patients with acute myeloid leukemia (AML) (72). A population-based observational study found that individuals with diabetes have a modest but significantly increased risk of hematological malignancy, including AML (adjusted HR 1.10 [95% CI 1.08–1.12] *P* < 0.0001), and that diabetes is an independent risk factor for all-cause and cause-specific mortality (91). It remains unclear whether D2D3 production by activated neutrophils can induce abnormal proliferation of immature myeloid cells in the bone marrow, or if D2D3 is produced by immature bone marrow cells, as seen previously with suPAR (22), potentially contributing to both diabetes and AML. The source of D2D3 in the CKD population also remains unknown.

The ability of suPAR and its related proteins to cause a multiorgan injury has been further supported by a comprehensive proteomics analysis of 2,920 plasma proteins from 53,026 participants in the UK Biobank (92). Elevated suPAR levels were linked to 18 diseases spanning six organ systems, including seven liver conditions (e.g., fibrosis and cirrhosis), respiratory disorders, and CKD. Together, these findings underscore the diverse and multifactorial mechanisms through which uPAR and its circulating proteins contribute to multiple organ damage.

## suPAR and CV diseases

Chronic inflammation is a key driver of atherosclerosis, arterial thickening due to plaque buildup. This plaque, composed of fats, cholesterol, waste, calcium, and fibrin, forms due to factors like high blood pressure, cholesterol, and blood sugar. Atherosclerosis heightens the risk of clots, heart attacks (myocardial infarction), strokes, and aneurysms. Initially recognized as a biomarker of adverse CV outcomes, including stroke and myocardial infarction (93–96), suPAR is now identified as a direct mediator linking chronic inflammation, vascular dysfunction, and CV disease progression.

Multiple cohort studies show strong links between elevated suPAR levels, subclinical atherosclerosis, and future CV events. suPAR outperforms traditional inflammatory markers, like hsCRP and IL-6, in predicting atherosclerosis, myocardial infarction, and heart failure (97, 98), suggesting distinct pathogenic pathways. In a study of 2,273 individuals, suPAR was strongly associated with endothelial dysfunction and atherosclerosis, while CRP correlated mainly with adiposity, reflecting different inflammatory processes (99). Unlike CRP and IL-6, suPAR remains stable during acute events like myocardial infarctions and shows minimal circadian variation (100). The Multi-Ethnic Study of Atherosclerosis (MESA) found that baseline suPAR levels independently predicted coronary artery calcification progression in 5,500 participants, regardless of ethnicity, kidney function, or other risk factors (37). In individuals with atherosclerosis, a two-fold increase in suPAR raised CV event risk by 1.46, while levels higher than 3.0 ng/mL increased risk by 1.77 compared with those with suPAR levels below 2.0 ng/mL, even after adjusting for traditional risk factors and biomarkers, demonstrating suPAR’s additive prognostic value (37).

Genetic evidence further strengthens the case for a causal relationship between suPAR and atherosclerotic disease. A GWAS meta-analysis of over 24,000 individuals identified two *PLAUR* missense variants (rs4760 and rs2302524) associated with elevated suPAR levels, with rs4760 experimentally validated in vitro and in vivo (37). Mendelian randomization analyses using rs4760 revealed that each standard deviation increase in genetically predicted suPAR corresponded to higher odds of coronary artery disease (CAD) (55%), myocardial infarction (75%), and peripheral arterial disease (71%). Conversely, rare *PLAUR* variants appear protective against coronary disease through reduced suPAR levels (37). Crucially, the cardiovascular Mendelian-randomization estimates replicated with concordant direction and magnitude in two independent trans-ancestry datasets, the Million Veteran Program (≈ 180,000 CAD cases) and CARDIoGRAMplusC4D (≈ 185,000 CAD events), fulfilling the Bradford-Hill criterion of consistency (37).

Both membrane-bound uPAR and suPAR are thought to play a role in multiple stages of atherogenesis by influencing cell adhesion, migration, and signaling through complex interactions (Figure 3C) (101–103). The current working hypothesis suggests that in early atherosclerosis, endothelial dysfunction triggers local suPAR production, as evidenced by significantly higher coronary suPAR production in patients with isolated microvascular endothelial dysfunction (30.9 ng/min vs. –9.6 ng/min; *P =* 0.026) (104). Notably, suPAR release increases when mononuclear cells or thrombocytes are cultured with endothelial cells, underscoring the role of the endothelial layer in regulating suPAR release (105). In a murine model of atherosclerosis initiated by the expression of proprotein convertase subtilisin/kexin type 9 (PCSK9), elevated circulating LDL cholesterol levels led to larger atherosclerotic plaques (1.55 mm³ vs. 0.90 mm³) and necrotic cores in the aortic root (0.18 mm³ vs. 0.05 mm³), along with increased inflammatory infiltration (Mac2 staining: 47.3% vs. 27.6%) in mice with higher circulating suPAR levels compared with wild-type controls (37). Despite similar cholesterol levels in both models, this study indicated that elevated circulating suPAR concentrations both prime and promote plaque formation.

Consistent with suPAR-overexpression studies, genetic deletion of uPAR attenuates lesion formation: *Plaur*^–/–^ LDLR^–/–^ double-knockout mice fed a Western diet developed approximately 35% smaller aortic-root plaques with reduced macrophage and smooth-muscle content compared with uPAR-sufficient littermates, attributed to impaired monocyte adhesion and VCAM-1 expression (106). This protective effect, combined with an accelerated phenotype due to suPAR overexpression, demonstrates bidirectional causality.

Several mechanisms have been proposed by which suPAR may contribute to plaque development (Figure 3C): (a) Elevated circulating suPAR levels may stimulate injured aortas to secrete higher levels of CCL2, a key monocyte chemoattractant in atherosclerosis. (b) Increased suPAR may lead to a doubling of monocyte numbers during the early stages of disease. (c) It may promote proinflammatory phenotypes in both circulating monocytes and bone marrow–derived macrophages, characterized by increased CCR2 and CX3CR1, decreased MHCII, and reduced membrane-bound uPAR. Those changes are associated with enhanced monocyte recruitment, impaired regulatory T cell activation, and heightened phagocytosis and efferocytosis. (d) Membrane-bound uPAR on endothelial cells may facilitate leukocyte adhesion and transmigration via activation of αvβ3 integrin, contributing to vascular remodeling and atherogenesis (37). Whether all or any of these pathways are essential in driving atherosclerosis remains to be further tested; however, strong genetic and experimental evidence support suPAR’s role in CV disease, making it a promising target for prevention and treatment.

## Summary

The kidneys and the innate immune system share complex, bidirectional interactions that extend beyond filtration and excretion. In CKD, dysregulated innate immune responses, particularly involving macrophages, drive chronic inflammation and accelerate kidney damage. Declining kidney function leads to uremic toxin accumulation, impairing immunity and increasing susceptibility to infections and CV diseases. This systemic immune dysregulation further exacerbates renal damage and contributes to CKD prevalence in inflammatory conditions like CV disease, diabetes, and HIV (107, 108). Indeed, while some kidney diseases, including IgA nephropathy and lupus nephritis, are rooted in autoimmunity, most arise from complex interactions between the kidneys and the immune system. In this context, the uPAR system, particularly its soluble forms such as suPAR and D2D3, has emerged as a key mediator linking inflammation, immunity, and coagulation (109, 110).

Here, we highlight the diverse and synergistic molecular mechanisms through which uPAR and its associated circulating proteins contribute to organ damage. Whether strategies such as lowering suPAR levels, removing the D2D3 protein, or blocking uPAR signaling in specific cell types, such as kidney cells or pancreatic β cells, represent viable therapeutic approaches (“druggable” pathways) for some or multiple indications remains to be determined. Diverse murine models indicate that pathogenic phenotypes can often be mitigated using pan anti-suPAR/D2D3 antibodies, emphasizing the potential for developing multimodal therapies that target multiple pathways across a range of conditions. Although no therapies are currently available that effectively lower suPAR levels, novel approaches involving monoclonal antibodies are currently being investigated. WAL0921, a first-in-class anti-suPAR monoclonal antibody developed by Walden Biosciences, is currently in ongoing phase II studies in glomerular kidney diseases (ClinicalTrials.gov NCT06466135), aiming to directly test the therapeutic potential of targeting this pathway. As our understanding of signaling roles of uPAR and its associated proteins grows, their impact is likely to extend well beyond kidney diseases, offering new opportunities in diverse areas of human health.

## Funding support

This work is the result of NIH funding, in whole or in part, and is subject to the NIH Public Access Policy. Through acceptance of this federal funding, the NIH has been given a right to make the work publicly available in PubMed Central.

- NIH grants R01 DK132072 (to JR), R01 DK133364 (to SS), R01HL153384 (to SSH).

## Version 1 01/02/2026

Electronic publication
